# Construction and Analysis of the Complete Genome Sequence of Leprosy Agent Mycobacterium lepromatosis

**DOI:** 10.1128/spectrum.01692-21

**Published:** 2022-04-25

**Authors:** Francisco J. Silva, Diego Santos-Garcia, Xiaofeng Zheng, Li Zhang, Xiang Y. Han

**Affiliations:** a Institute for Integrative Systems Biology (I2SysBio), University of Valencia and CSIC, Paterna, Spain; b Genomics and Health Area, Foundation for the Promotion of Sanitary and Biomedical Research, Valencia, Spain; c Laboratory of Biometry and Evolutionary Biology UMR CNRS, University of Lyon, Villeurbanne, France; d Department of Bioinformatics and Computational Biology, The University of Texas MD Anderson Cancer Center, Houston, Texas, USA; e Department of Laboratory Medicine, The University of Texas MD Anderson Cancer Center, Houston, Texas, USA; Institute of Bioinformatics and Applied Biotechnology

**Keywords:** *Mycobacterium leprae*, *Mycobacterium lepromatosis*, reductive evolution, genomics, leprosy

## Abstract

Leprosy is caused by Mycobacterium leprae and Mycobacterium *lepromatosis*. We report construction and analyses of the complete genome sequence of *M. lepromatosis* FJ924. The genome contained 3,271,694 nucleotides to encode 1,789 functional genes and 1,564 pseudogenes. It shared 1,420 genes and 885 pseudogenes (71.4%) with M. leprae but differed in 1,281 genes and pseudogenes (28.6%). In phylogeny, the leprosy bacilli started from a most recent common ancestor (MRCA) that diverged ~30 million years ago (Mya) from environmental organism Mycobacterium haemophilum. The MRCA then underwent reductive evolution with pseudogenization, gene loss, and chromosomal rearrangements. Analysis of the shared pseudogenes estimated the pseudogenization event ~14 Mya, shortly before species bifurcation. Afterwards, genomic changes occurred to lesser extent in each species. Like M. leprae, four major types of highly repetitive sequences were detected in *M. lepromatosis*, contributing to chromosomal rearrangements within and after MRCA. Variations in genes and copy numbers were noted, such as three copies of the gene encoding bifunctional diguanylate cyclase/phosphodiesterase in *M. lepromatosis*, but single copy in M. leprae; 6 genes encoding the TetR family transcriptional regulators in *M. lepromatosis*, but 11 such genes in M. leprae; presence of *hemW* gene in *M. lepromatosis*, but absence in M. leprae; and others. These variations likely aid unique pathogenesis, such as diffuse lepromatous leprosy associated with *M. lepromatosis*, while the shared genomic features should explain the common pathogenesis of dermatitis and neuritis in leprosy. Together, these findings and the genomic data of *M. lepromatosis* may facilitate future research and care for leprosy.

**IMPORTANCE** Leprosy is a dreaded infection that still affects millions of people worldwide. Mycobacterium *lepromatosis* is a recently recognized cause in addition to the well-known Mycobacterium leprae. *M. lepromatosis* is likely specific for diffuse lepromatous leprosy, a severe form of the infection and endemic in Mexico. This study constructed and annotated the complete genome sequence of *M. lepromatosis* FJ924 and performed comparative genomic analyses with related mycobacteria. The results afford new and refined insights into the genome size, gene repertoire, pseudogenes, phylogenomic relationship, genome organization and plasticity, process and timing of reductive evolution, and genetic and proteomic basis for pathogenesis. The availability of the complete *M. lepromatosis* genome may prove to be useful for future research and care for the infection.

## INTRODUCTION

Leprosy is one of the oldest known human diseases. Despite tremendous advances during the last several decades, this infection continues to be an important health problem in many developing countries. Leprosy affects skin and peripheral nerves chronically and manifests in the clinical forms of tuberculoid, borderline, lepromatous, and diffuse lepromatous leprosy (DLL) (1, 2). Since initial discovery in 1873, Mycobacterium leprae has been known to be the sole cause of leprosy (3). In 2008, a new species, named Mycobacterium *lepromatosis*, was found to be a second cause of leprosy in two patients of Mexico origin who died of DLL (4).

Genome sequencing of M. leprae has revealed a phenomenon of reductive evolution (5). The genome size (3.27 Mb) was much smaller (>1 Mb) than that of related Mycobacterium tuberculosis (6). More surprisingly, it contained around 1,600 pseudogenes with loss of ~50% of the ancestral genes (7). Comparative analyses of M. leprae pseudogenes with their orthologous M. tuberculosis genes have led to the proposal that a massive pseudogenization took place ~20 million years ago (Mya) (7). The lean M. leprae genome has been extraordinary stable, as revealed by genome sequences and typing of many worldwide strains that showed only clonal differences (0.005%) (8).

The clonal differences among M. leprae strains contrast a 9.1% genetic difference with *M. lepromatosis* that was revealed through analysis of 20 genes and pseudogenes (22.8 kb) (9). The study also estimated a divergence time of ~10 Mya between the two leprosy bacilli. With analysis of the draft genome of *M. lepromatosis* strain Mx1-22A, this divergence was refined to 13.9 Mya (10). The leprosy bacilli are phylogenetically closely related to Mycobacterium haemophilum (11), an environmental organism with very low pathogenicity. The genome sequence of M. haemophilum (4.24 Mb) shows that almost all coding genes (CDS) are functional in contrast to the decayed genomes of the leprosy bacilli (12).

Clinical and pathological studies on *M. lepromatosis* have been revealing as well. Many studies have identified this agent in patients with leprosy from American and Asian countries, including Mexico (4, 13, 14), Canada (15), Costa Rica (16), Brazil (17), Myanmar (17), and Singapore (18). Some of these studies have also revealed dual agent infections caused by *M. lepromatosis* and M. leprae in some patients (14, 16–18). In Mexico, *M. lepromatosis* is likely the dominant cause of leprosy and specific for the endemic DLL (14). DLL is a unique and severe form of lepromatous leprosy occurring in ~20% of patients (9, 14). It is characterized by diffuse nonnodular cutaneous infiltration and recurrent crops of large and sharply demarcated ischemic skin lesion called Lucio’s phenomenon (2, 19). In the advanced stage, lesions may become ulcerated or even generalized, particularly on the lower extremities, leading to fatal secondary infection and sepsis. Pathologically, the mycobacteria invade deep into the subcutis, blood vessels, and internal organs, in addition to skin and nerves – the hallmark of leprosy (14, 20). The vasculitis eventually leads to endothelial proliferation, vascular occlusion, ischemia, and skin necrosis (4, 14). The discovery and further characterization of *M. lepromatosis* should thus enable research and insight into the prominent involvement of subcutis (panniculitis) and blood vessels (leukocytoclastic vasculitis) in DLL.

In Europe where leprosy has long been eliminated, red squirrels on British Isles have been found to be infected with the leprosy bacilli (21). The study further showed that, while the squirrel M. leprae strains were most closely related to the medieval human strains, the squirrel *M. lepromatosis* strains had a divergence time of 27,000 years from the Mexican strain Mx1-22A. This divergence time raises an intriguing question as to how the bacterium landed in the British red squirrels.

While the draft genome of *M. lepromatosis* Mx1-22A has characterized the vast majority of genes and pseudogenes (10), the presence of 126 contigs and potential omit of unique component make it desirable to construct and analyze the complete genome sequence of this organism. Here, we report construction and annotation of the complete genome sequence of *M. lepromatosis* strain FJ924 and comparative genomic analyses with related mycobacteria. This study has improved our understanding of the complete evolution of the chromosome of both species, including rearrangements, gene losses and gene duplications. We have also estimated the relative ages of pseudogenes from M. leprae and *M. lepromatosis* to time the process of massive pseudogenization in relation to the divergence points.

## RESULTS AND DISCUSSION

### Construction of the complete genome.

The construction of *M. lepromatosis* genome encountered several technical hurdles. Due to the lack of axenic cultivation, the quantity and quality of genomic DNA became the first rate-limiting step. The genomic DNA was extracted from a dried, stained, and archived smear slide, with only ~18% of sequenced reads (12.3 of 69 million reads) belonging to *M. lepromatosis.* Contaminant reads included human host DNA and nonspecific bacteria DNA. Some bacterial DNA reads, from Thermus scotoductus particularly, were likely introduced and/or amplified during the library preparation step that used polymerase chain reactions (PCR) to boost target quantity. Other nonspecific bacterial DNA, such as from Propionibacterium acnes, Micrococcus luteus, and others, were likely from the stained smear that used regular reagents for acid-fast stain, tap water, and microscopy immersion oil. To overcome contamination, initial steps used matches to the nearest M. leprae genome to capture specific reads, *de novo* assembly, systematic tagging and removal of contaminant contigs, and alignment to M. leprae for draft construction. Later more iterative cycles of mapping, *de novo* assembly, and gap filling were used to retrieve unique sequences, close gaps and refine drafts. PCR and Sanger sequencing of amplicons were used to close final gaps, verify assembly of several long contigs, and settle copy numbers of short repetitive sequences. The genome coverage was 525×.

### Genome features and phylogeny.

The seamless genome of *M. lepromatosis* strain FJ924 contained 3,271,694 bp. The genome encoded 1,789 CDS, 1,564 pseudogenes, and 63 noncoding RNA genes (3 rRNA, 46 tRNA, 1 tmRNA and 13 other ncRNA) (Table 1). It was slightly longer, by 3,623 bp (0.1%), than the genome of M. leprae Br4923 (3,268,071 bp), but much shorter, by 964,071 bp (29.5%), than the M. haemophilum genome (4,235,765 bp). The CDS covered 50.4% of the genome and averaged 922 bp per CDS. The pseudogenes covered 36.4%, averaging 761 bp per pseudogene. Intergenic sequences, including repetitive elements, covered the remaining 13.2%, averaging 129 bp per intergenic spacer.

**TABLE 1:** Comparative genomic features of *M. lepromatosis* FJ924 and related mycobacteria^*a*^

Feature	*M. lepromatosis* strain		M. leprae strain		M. haemophilum DSM 44634	*M. uberi*s Jura	M. tuberculosis H37Rv
	FJ924	Mx1-22A	Br4923	TN			
Genome size (bp)	3,271,694	3,206,741	3,268,071	3,268,203	4,235,765	3,122,721	4,411,532
Protein coding genes (CDS)	1,789	1,477	1,604	1,605	3,728	1,759	4,018
CDS pseudogenes	1,564	1,331	1,116	1,115	153	1,081	13
rRNA genes	3	3	3	3	3	2	3
tRNA genes	46	45	45	45	45	44	45
tmRNA gene	1	1	1	1	-	1	1
Other noncoding RNA genes	13	3	3	2	3	3	30
% GC content	57.89	57.89	57.80	57.80	63.90	57.50	65.60
No. contigs	1	126	1	1	1	54	1

a Data from GenBank/RefSeq genome. *M. lepromatosis* FJ924, this work.

**FIG 1:**
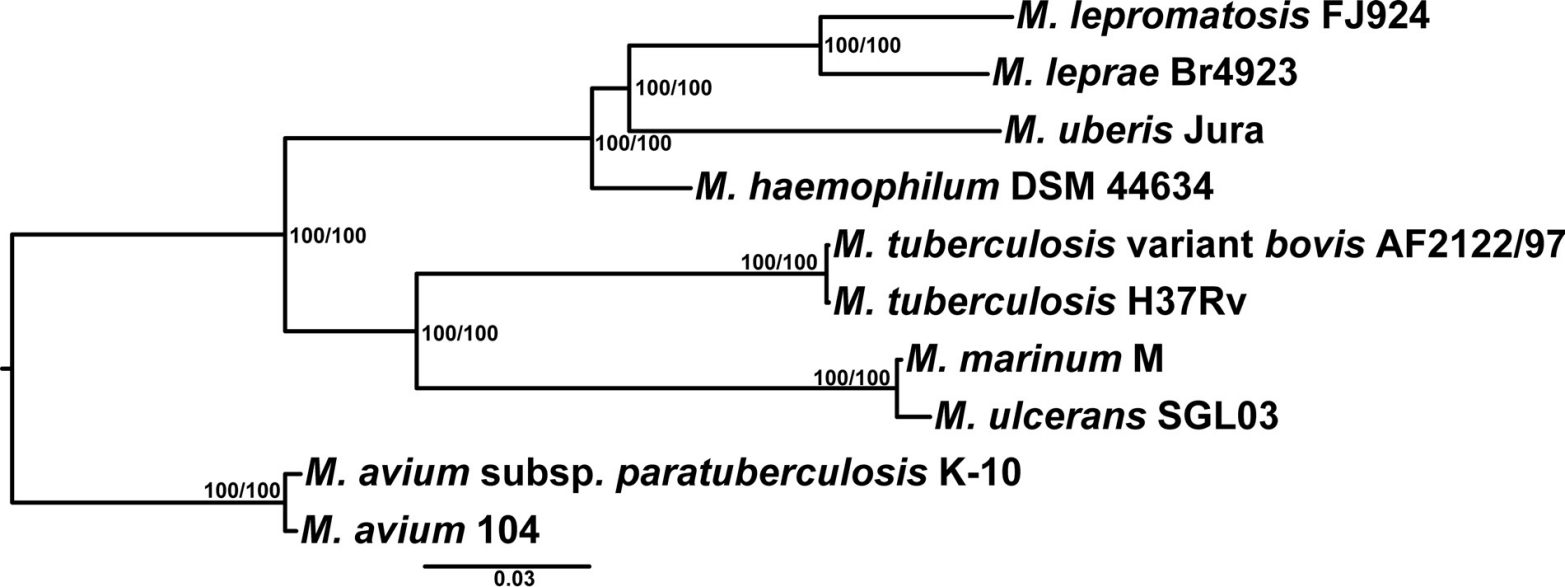
Phylogenomic tree of selected mycobacterial species. ML tree was inferred using a concatenated conserved protein alignment (316,716 amino acid positions) under a JTT+F+R3 substitution model. Support values were obtained with 1000 ultrafast bootstraps (right node labels) and 1000 SH-aLRT (left node labels).

A phylogenomic tree was constructed based on the proteomes (316,716 alignment sites) of well-known mycobacteria and the newly named and sequenced Mycobacterium *uberis*, a yet-to-be cultivated organism that causes nodular thelitis and tuberculoid scrotitis in cows and goats (22). As shown in Fig. 1, M. leprae and *M. lepromatosis* stem from a most recent common ancestor (MRCA) that forms a clade with M. haemophilum and *M. uberis*. Shortly after the divergence of M. haemophilum, the lineages of *M. uberis* and the MRCA diverged again and both underwent genome downsizing, pseudogenization, and decrease in GC content (Table 1).

### Comparison with the draft genome of *M. lepromatosis* Mx1-22A.

The FJ924 genome was compared with the draft genome of *M. lepromatosis* Mx1-22A that consisted of 3,206,741 bp from 126 contigs (10). This detected 66 kb extra nucleotides from 56 segments with lengths >300 bp, with the largest segment covering 8,518 bp (Table S1A). Annotation of these segments revealed 27 CDS (21 kb, 1.5% of all CDS) and 65 pseudogenes (44 kb, 4.2% of all pseudogenes). Most of these genes and pseudogenes were absent in Mx1-22A, while a few of them showed partial sequence of the annotated feature (Table S1B and S1C). Shorter Mx1-22A was likely due to loss of some *M. lepromatosis* unique sequences during array capture to the M. leprae genome, to the assembler that discards/collapses repetitive regions, and to low sequencing coverage of the genome (55×) (10).

Single nucleotide polymorphisms (SNPs) between Mx1-22A and FJ924 were identified with MAUVE (23, 24). After exclusion of ambiguities and inaccuracies near the edges of Mx1-22A contigs, a final set of 11 SNPs were obtained, with six in genes (five in CDS and one in the 16S rRNA gene) and five in intergenic regions (Table S2). Four SNPs detected in coding genes produced one amino acid difference between the proteins encoded by the two strains. The fifth SNP in a CDS encoded a PPE protein of 410 amino acids in FJ924 (MLPF_1455), but it caused a stop codon in Mx1-22A, hence a pseudogene (MLPM_1054). The limited number of SNPs suggests that the divergence between Mx1-22A and FJ924, strains likely from the Monterrey region in north central Mexico and the Sonora region in north western Mexico, respectively (~1000 km away) (4, 10), was very recent, probably a few hundred years ago. Similarly, another *M. lepromatosis* strain PL-02, also from Mexico, diverged from Mx1-22A ~186 years ago (21).

Among the extra CDS detected, the complete FJ924 genome contained two tandemly arranged asparagine permease genes (MLPF_1849 and 1850), like M. leprae, and three identical copies of the gene encoding bifunctional diguanylate cyclase/phosphodiesterase, unlike M. leprae, *M.uberis* and M. tuberculosis with a single copy (further analysis later). The Mx1-22A contained single asparagine permease gene and single diguanylate cyclase/phosphodiesterase gene (10); such omissions were likely due to relaxed genome assembly and/or lack of copy count instead of true absence. Assembly errors in three Mx1-22A contigs were also seen, such as contig-111, a 14,151-bp fragment, that showed a minus orientation of the first 8,706 bp.

### Repetitive sequences.

**FIG 2:**
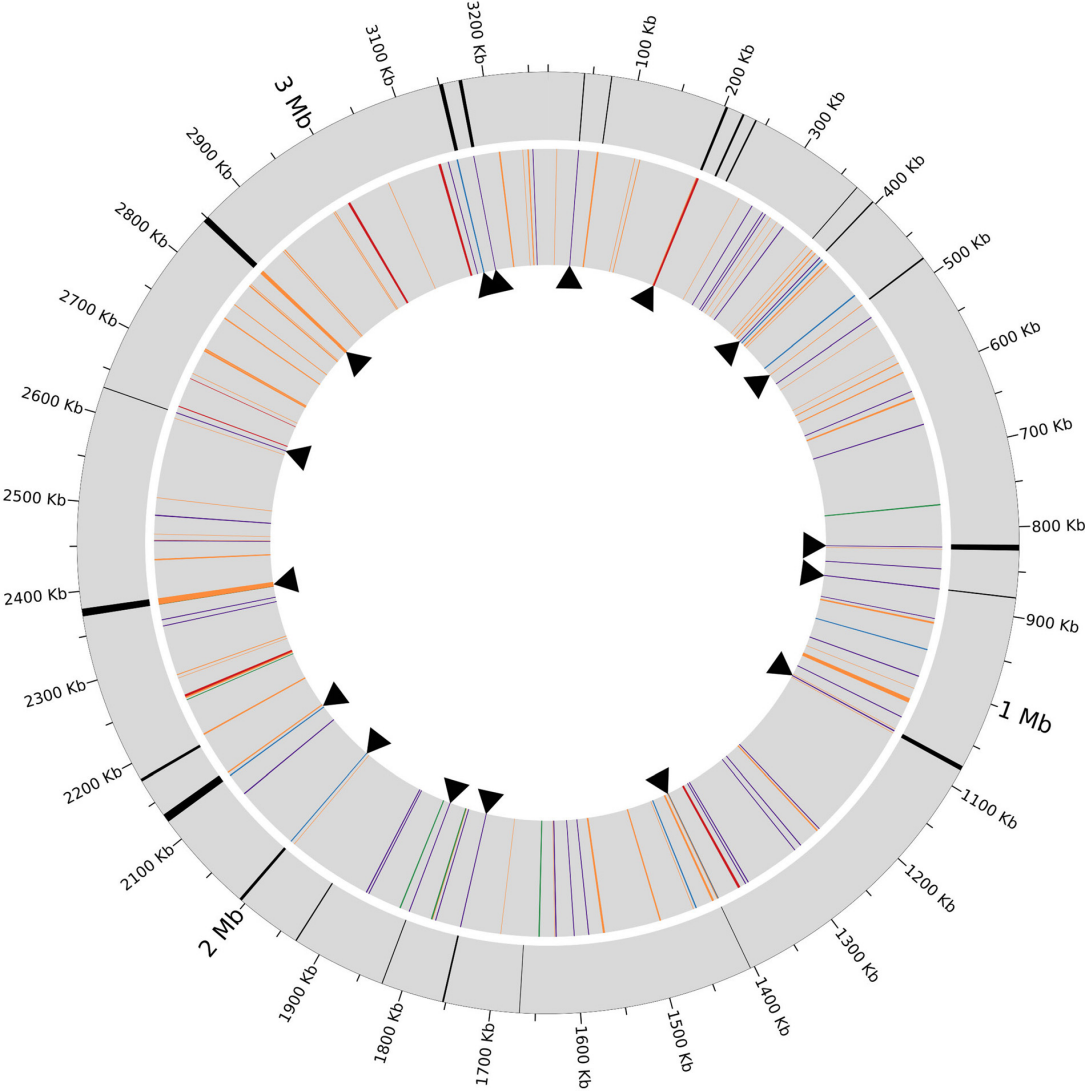
Synteny breaks and repeat sequences identified in *M. lepromatosis* FJ924. The outside circle displays the 24 synteny breaks (black) detected in the genome comparing with M. leprae. The inner circle displays the positions of repeat sequences: RLPM (purple), LPMREP (red), REPLPM (blue), LPMRPT (yellow) repeat families, and other unidentified repeats (orange). Arrowheads mark the coincidences between the positions of breaks and repeat sequences.

The M. leprae genome is characterized by the presence of numerous repetitive sequences that likely play a role in genome plasticity (25). Repetitive sequences that appeared twice or more in the *M. lepromatosis* FJ924 genome were analyzed (Table S3). In total, they accounted for 3.5% of the genome and were scattered in 125 genome segments. Some repeats dispersed similarly to the repetitive elements in the M. leprae genome (25). Four main families of repetitive elements were identified: types A, B, C and D, with 38, 8, 8 and 6 copies, respectively (Fig. 2 and Table S3A). These repeats were named as RLPM, LPMREP, REPLPM, and LPMRPT to correspond to RLEP, LEPREP, REPLEP, and LEPRPT, respectively, in the M. leprae genome.

Among the 38 copies of RLPM of various extension lengths, a core sequence of 626 nucleotides was identified in 18 copies (see consensus in Table S3B). The complete or partial core sequences were highly conserved in the 38 copies (99.2–100% identity). However, compared with all 37 RLEP copies in M. leprae, RLPM showed low sequence identity, in 25 copies at best 75% of 126 nucleotides with several gap sites. Nonetheless, in view of the nearly identical copy numbers, similar average length (~700 bp), and some homologous genome locations, RLEP and RLPM probably evolved from the same ancestral repeat after divergence from MRCA.

Type B repeats LPMREP resembled the LEPREP repeats in M. leprae in BLAST matches (79–83%) and copy numbers. Both showed five complete copies and three fragment copies in their respective genomes. The origin of these repeats seems to be a pseudogene of a putative group II intron maturase-related protein, which persisted from the MRCA. Based on the alignment of the complete copies of LPMREP, a core sequence of 2,453 nucleotides was identified (Table S3B). Recently, these repeats were used in a *M. lepromatosis* molecular diagnostic assay, but under the abbreviation of ‘RLPM’ (16).

Type C repeats REPLPM in *M. lepromatosis* were equivalent to the REPLEP repeats in M. leprae, but with gapped alignments and an overall 74% match. The copy numbers also varied: 8 copies for REPLPM but 15 copies for REPLEP (25). The REPLPM copies displayed different lengths but a consensus core of 1,123 nucleotides could be inferred (based on 50% coverage). This core sequence (Table S3B) was completely included in REPLPM_07.

Type D repeats LPMRPT were equivalent to LEPRPT repeats in M. leprae with 87% similarity covering almost the complete sequence. Only four of LPMRPT repeats were large (~1,200 bp) (see LPMRPT_01 in Table S3B), while the two others were short fragments.

Functionally, the four repetitive elements likely also played a role in genome plasticity and organization (see below). Other repetitive sequences were also annotated (Table S3A). They likely arose from segmental duplications or gene families.

### Comparative genomics: synteny and proteome.

**FIG 3:**
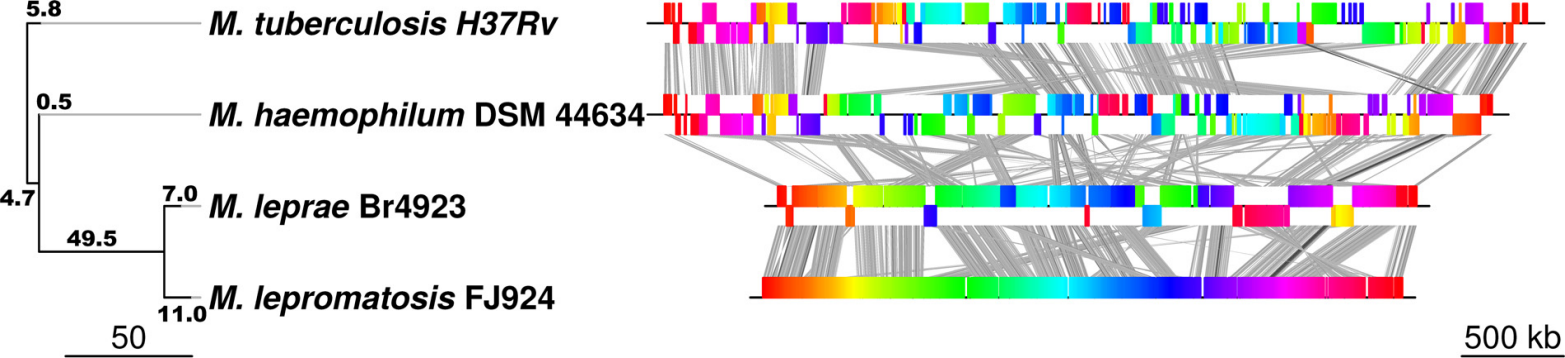
Synteny among mycobacterial genomes. (Left) Genome rearrangement phylogeny obtained with the Neighbor joining method and a distance matrix showing the minimal number of inversion events required to explain the differences between any pair of genomes obtained with GRIMM. Branch lengths are the numbers of inversion events estimated with the phylogenetic method. (Right) Graphic linear representation of the genome rearrangements observed comparing the four mycobacterial genomes. The graph was obtained with Mauve and displayed with genoPlotR.

Synteny among the genomes of M. leprae, *M. lepromatosis*, M. haemophilum and M. tuberculosis was compared with the program MAUVE (23) (Fig. 3). Many differences were observed between the two leprosy bacilli, by one way, and between M. haemophilum and M. tuberculosis, by the other, indicating events of chromosomal rearrangement.

To infer the minimal number of chromosomal rearrangements among them, a pairwise inversion distance matrix was constructed with the program GRIMM (26), which predicted 18 inversion events between M. leprae and *M. lepromatosis* and 11 between M. haemophilum and M. tuberculosis (Fig. 3). In addition, the MRCA of leprosy bacilli incurred ~50 chromosomal rearrangements after divergence from M. haemophilum. Comparisons of the consecutive CDS and pseudogenes in M. leprae and *M. lepromatosis* further rendered 25 syntenic blocks as the likely outcome of major rearrangement events (Table S4). These blocks and the 24 synteny breaks were analyzed for genesis by comparing them with ancestral M. haemophilum and M. tuberculosis (Table S5). For example, blocks 4 and 5, and blocks 21 and 22 in *M. lepromatosis* was similarly continuous as in M. haemophilum and M. tuberculosis whereas they were separated in M. leprae. Similarly, by the order of M. leprae, seven colinear blocks boundaries aligned with M. haemophilum (and with M. tuberculosis in six cases) to suggest ancestral origin. Together, these seven M. leprae connections affected 11 synteny breaks in *M. lepromatosis* (Table S5).

At the 24 synteny breaks, 17 repetitive elements were noted to be involved: 9 RLPM, 3 REPLPM, 2 LPMREP and 3 other repeats (Fig. 2 and Table S5). These observations reveal the abrupt effect of repeats through recombination to shape the genome of *M. lepromatosis*. These chromosomal rearrangements may also produce gene duplications and deletions and affect gene expression. Similar effects were also previously observed for the genome of M. leprae (25).

**FIG 4:**
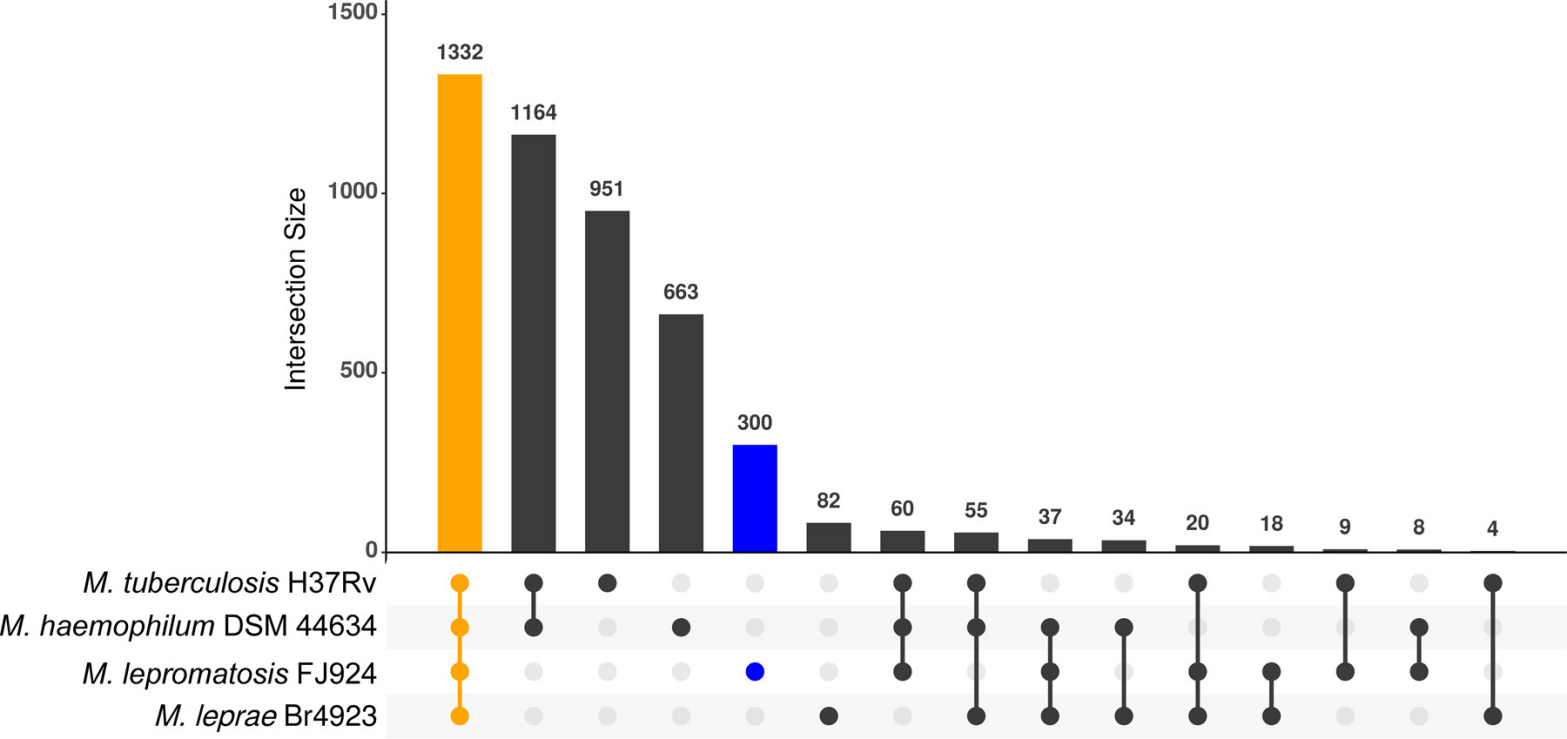
Comparative analyses of mycobacterial proteomes. UpSet plot showing the computed coding gene clusters (intersections) derived from the proteomes encoded in the complete genomes of *M. lepromatosis* FJ924, M. leprae Br4923, M. haemophilum DSM44634, and M. tuberculosis H37Rv. Shared coding gene clusters (core) and *M. lepromatosis* FJ924 specific coding gene clusters are highlighted in yellow and blue, respectively.

Orthologous clusters of proteins were estimated with the program OrthoFinder v2.3.3 (27) for proteomes of the four mycobacteria (Fig. 4). A core proteome of 1,332 protein clusters, with at least one member encoded in each genome, was detected. Of them, a set of 1,227 clusters included a single protein in each proteome (single copy orthogroups). The number of clusters with only proteins from one leprosy proteome was relatively high (82 in M. leprae and 300 in *M. lepromatosis*). However, most of them were annotated as hypothetical proteins, only a few with an annotated function (13 in *M. lepromatosis*). There were also several orthogroups, including proteins from only leprosy proteomes (18 clusters). Among the latter, three showed a functional annotation: MLPF_0519 (LamB/YcsF family protein), MLPF_0609 (PPE family protein) and MLPF_0662 (lysophospholipase protein domain). In summary, the proteome of *M. lepromatosis* is a reduced version of cultivable mycobacteria with almost no specific functional proteins.

### History of pseudogenization in the leprosy bacilli.

The pseudogenization events in *M. lepromatosis* and M. leprae, in terms of the ages of pseudogenes, were analyzed to time the occurrences, i.e., before divergence, after divergence, or both. A set of 355 shared pseudogenes, 24 unique pseudogenes in *M. lepromatosis*, and 30 unique pseudogenes in M. leprae were obtained for analysis through annotation and alignment (>200 nucleotide sites) of orthologous functional CDS in M. haemophilum and M. tuberculosis and CDS or pseudogene in one leprosy bacillus. While the requirement of orthology precluded all pseudogenes for analysis, the qualified ones should render insights into the tempo of pseudogenization.

**FIG 5:**
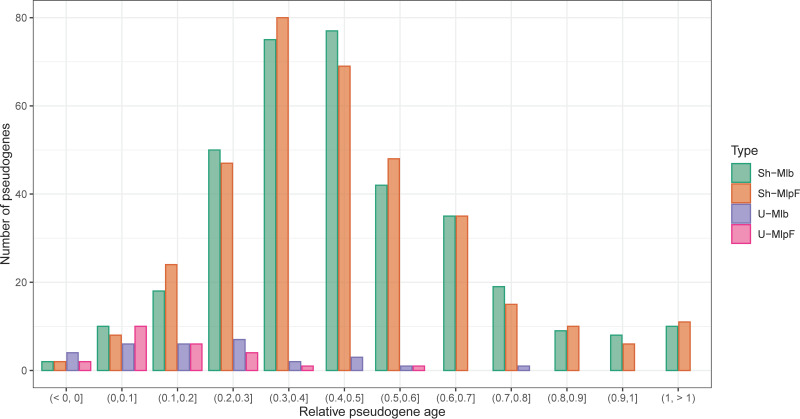
Relative ages of pseudogenes. Histogram with the relative ages estimated for four types of pseudogenes. A value of 1 corresponds to the time of divergence between M. haemophilum and the leprosy bacilli. A value of 0 is present time. From left to right: M. leprae shared pseudogenes (with an orthologous pseudogene in *M. lepromatosis*, green color), *M. lepromatosis* shared pseudogenes (with an orthologous pseudogene in M. leprae, orange color), M. leprae unique pseudogenes (with a CDS annotated in *M. lepromatosis*, blue color) and *M. lepromatosis* unique pseudogenes (with a CDS annotated in M. leprae, pink color).

The relative ages of pseudogenes (*Rpa*) are shown in Fig. 5. Values for unique pseudogenes were small, with mean and SD of 0.138 ± 0.139 and 0.216 ± 0.190 for *M. lepromatosis* and M. leprae, respectively, suggesting more recent occurrences after divergence. Values for shared pseudogenes were larger, 0.462 ± 0.238 for *M. lepromatosis* and 0.467 ± 0.254 for M. leprae along with a wider and overlapping distribution. An x-y plot of the shared pseudogenes showed a positive correlation (R^2^= 0.704) (data not shown), indicating a common pseudogenization process prior to divergence. Together, the analysis of *Rpa* suggests that most of them occurred approximately during the same period as an event of massive pseudogenization.

**FIG 6:**
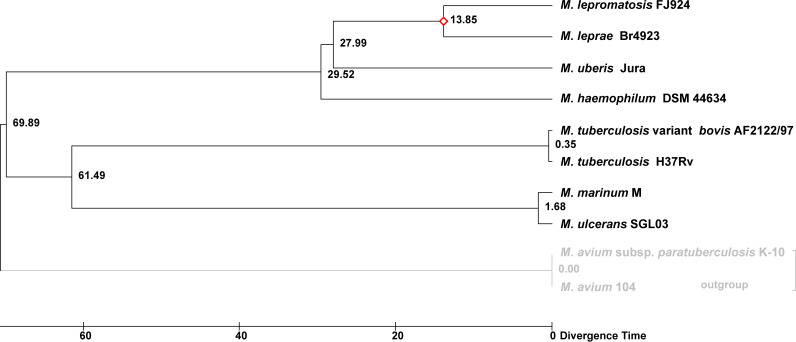
Divergence times of mycobacterial species. A timetree analysis using the RelTime method with a concatenated alignment (316,716 sites amino acid sites) was used. Evolutionary model was JTT+G+I+F. The calibration node is marked (red). Divergence time in millions of years ago.

A time tree analysis using the RelTime method (28) estimated the time of divergence between M. haemophilum and the MRCA of the leprosy bacilli to be 29.52 Mya (Fig. 6). When this value was set to be *Rpa* of 1, Mya values for the shared pseudogenes were 13.64 ± 7.03 for *M. lepromatosis* and 13.79 ± 7.5 for M. leprae. This result and the positive correlation between the values of shared pseudogenes suggest a massive pseudogenization event occurred close but slightly prior to divergence of the two leprosy bacilli at 13.85 Mya, noted previously (10), but much later than the divergence from the related species *M. uberi*s at 28 Mya (Fig. 6).

### Pathogenetic insights from shared genomic features.

The leprosy bacilli have a moderate pathogenicity, between rarely pathogenic M. haemophilum and highly pathogenic M. tuberculosis (11). The lean genomes and shared genomic features underscore the clinical and pathological features of leprosy, i.e., long incubation period, insidious onset, dermal and neuronal invasion of bacillus-laden macrophages, and chronicity, contrasting to mainly pulmonary infection in tuberculosis.

Table 2 shows the numbers of shared and unique CDS and pseudogenes for M. leprae and *M. lepromatosis*. The two bacilli shared 1,420 CDS and 885 pseudogenes (71.4% of genome) but differed in 1,281 CDS and pseudogenes (28.6%), although some of them may be associated with the different annotation protocols. Some shared features were examined to highlight common pathogenesis in leprosy.

**TABLE 2:** Numbers of shared and unique CDS and pseudogenes between *M. lepromatosis* and M. leprae

M. leprae Br4923	*M. lepromatosis* FJ924			Total
	CDS	Pseudogene	Absent	
CDS	1420	128	56	1604
Pseudogene	54	885	177	1116
Absent	315	551		866
Total	1789	1564	233	3586

Among all known mycobacteria (~160 species), neuronal invasion is unique to the leprosy bacilli. In M. leprae, adherence to Schwann cells seems to require the presence of the DNA-binding protein HU and the cell wall antigen phenolic glycolipid 1 (PGL-I) (29). PGL-I also subverts host immune cells to shield the bacillus from clearance (30). The synthesis of PGL-I requires 6 enzymes/genes (31). In *M. lepromatosis* FJ924, the PGL-I genes were identified as MLPF_0154, 0155, and 0156, and MLPF_2688, 2689, and 2690 in two clusters without related pseudogenes. The bacillus also contained the gene encoding DNA-binding protein HU (MLPF_0968) that is also known as mycobacterial DNA binding protein 1 or laminin-binding protein (32). This protein consists of an N-terminal HU-like region and a C-terminal IDR (intrinsically disordered region). The *M. lepromatosis* protein contained 195 residues, slightly smaller than the proteins of M. leprae, M. haemophilum and M. tuberculosis (200, 213 and 214 residues, respectively). The length difference was close to the C terminus at the IDR, essential region for such function as genome compaction or suppression of DNA synthesis (33).

Several ESX secretion systems (type VII secretion systems or early secretory antigenic target [ESAT6] secretion) are important for virulence of mycobacterial species (34, 35). Among them, ESX-1 system is the main determinant of virulence in M. tuberculosis. The genes encoding the proteins of the ESX-1 system are located in two or three clusters in the genomes of M. tuberculosis, M. haemophilum and *M. uberis* (22). In *M. lepromatosis* Mx1-22A and M. leprae, around a quarter of the genes are nonfunctional pseudogenes (10). In *M. lepromatosis* FJ924, the genes and pseudogenes of the ESX-1 system were located at two clusters in the genome, one from pseudogene MLPF_0041 (*espE*) to gene MLPF_0062 (*mycP*) (one of the three *mycP* genes of *M. lepromatosis*) and the other comprising genes MLPF_601, 602, and 603 (*espA*, *espC* and *espD*). Retention of some ESX-1 genes in the leprosy bacilli suggests some functionality of the secretion system.

The ESX-5 system plays a pivotal role in the secretion of proteins rich in Pro-Glu (PE proteins) and Pro-Pro-Glu (PPE proteins) (35). *M. lepromatosis* contained mainly the core genes of the system, MLPF_0876 to 0880 (*eccA*, *eccE*, *mycP*, *eccD* and *espG*) and MLPF_0889 and 0890 (*eccC* and *eccB*). A pseudogene of the esat-6 like protein (MLPF_0881) was detected between these two gene clusters. In the orthologous M. leprae region, neither an *esat-6* gene nor a pseudogene was detected. Genes encoding the PE/PPE proteins were examined in the genomes of leprosy bacilli for comparison with those in M. haemophilum and M. tuberculosis. OrthoFinder clustered these proteins/genes in 10 orthogroups (Table S6). While the M. haemophilum and M. tuberculosis genomes each contained 46 genes spread in the clusters, *M. lepromatosis* and M. leprae each contained only 13 genes but 34 and 32 pseudogenes, respectively (Table S7).

PE/PPE proteins are noted to be antigenic determinants in M. tuberculosis to play a role in immuno-pathogenesis (36). To the less virulent leprosy bacilli, these alarming molecules would be detrimental to the survival. Rather, in M. leprae, preferential inactivation of PE/PPE genes is noted whereas PGL-I and its genes, beneficial to the bacillus, are retained, which likely aid immune evasion and survival of the bacillus during ~20 million years of reductive evolution (11). In *M. lepromatosis*, these were also true, with statistical significance (Table 3). As a benchmark for gene retention, all 52 genes but one that encode ribosomal proteins, essential for ribosome structure, protein synthesis and bacterial survival, remained (Table 3). The same was true for M. leprae. The similar numbers of genes and/or pseudogenes for PE/PPE, PGL-I, and ribosomal proteins suggest their origins in the MRCA and persistence.

**TABLE 3:** Preferential inactivation of PE/PPE genes but retention of PGL-I genes and ribosomal protein genes in *M. lepromatosis* FJ924

Feature	PE/PPE comparison			PGL-I comparison			Ribosomal proteins		
	PE/PPE	All others	Total	PGL-I	All others	Total	RBS	All others	Total
No. genes	13	1776	1789	6	1783	1789	52	1737	1789
No. pseudogenes	34	1530	1564	0	1564	1564	1	1563	1564
Total	47	3306	3353	6	3347	3353	53	3300	3353
Inactivation odds ratio	3.04			0			0.02		
Statistical test	X^2^ = 12.6, *P* = 0.0004			Fisher’s *P* = 0.033			X^2^ = 43.3, *P* < 0.0001		

Dermal tropism is innate to the clade of M. haemophilum, lineages of leprosy bacilli, and *M. uberis*. Despite unknown environmental niches, M. haemophilum grows optimally at 30–32°C in culture and requires heme supplement (12, 37). The bacillus causes rare infections, mainly dermal and/or disseminated ones, in immunocompromised patients, such as long-term steroid users. In patients with leprosy, the most common sites of infection involve earlobes, extremities, nasal mucosa, and scrotum where the temperatures tend to be below 37°C to favor bacterial proliferation.

Finally, the large number of shared genes and pseudogenes would require a systematic approach to analyze their functional clusters and/or lack thereof. In view of the cultivation difficulty that has impeded the research for leprosy bacilli, future research endeavors in M. tuberculosis and M. haemophilum should help in this regard.

### Pathogenetic insights from unique genome features.

The two leprosy bacilli differed in CDS and pseudogenes, including 369 unique CDS in *M. lepromatosis* and 184 unique CDS in M. leprae (Table 2). Examination of these unique CDS and pseudogenes should offer insights into some varying pathological features in patients with each infection, such as more skin nodules associated with M. leprae but more invasion into subcutis, vessels and internal organs associated with *M. lepromatosis*.

**FIG 7:**
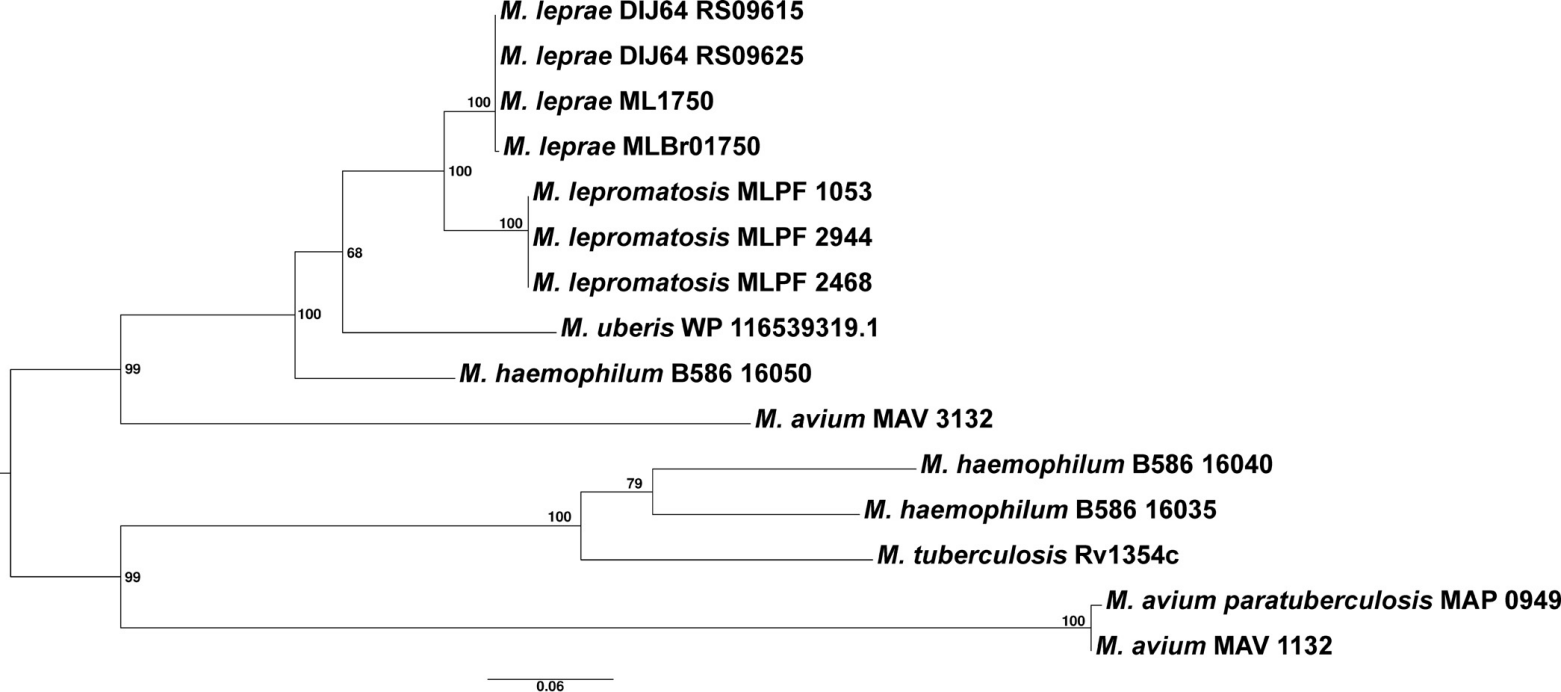
Phylogenetic reconstruction of diguanylate cyclase/phosphodiesterase genes in selected mycobacterial species. Maximum likelihood phylogeny obtained with the amino acid alignment (601 sites), using the evolutionary model JTT+G. Numbers at the nodes indicate bootstrap values obtained with 500 replicates.

The finding of three copies of the diguanylate cyclase/phosphodiesterase gene in *M. lepromatosis* (MLPF_1053, 2468 and 2944) contrasted the single copy status in M. leprae (MLBr01750), *M. uberis* (WP_116539319.1) and M. tuberculosis (Rv1354c) but resembled those in M. haemophilum. A phylogenetic reconstruction of the evolution of these proteins in several mycobacterial species (Fig. 7) revealed that the encoding gene was duplicated in the early ancestor of these species and Mycobacterium avium (taxonomy in Fig. 1). After this event, several gene gains and losses took place. *M. lepromatosis*, M. leprae and *M. uberis* retained the same copy and lost the other, in opposition to M. tuberculosis. Gene MLPF_1053 was located at a large syntenic block of > 200 kb (block 11, Table S4), suggesting ancestral origin. Gene MLPF_2468 arose through a 5,020-bp duplication involving MLPF_1053 and 1052 (a pseudogene derived from the transposase gene of an insertion sequence element) and the last part of pseudogene MLPF_1051. Gene MLPF_2944 was a duplicate of a 4,700-bp segment involving MLPF_2468 and the pseudogenes MLPF_2467 and 2466. In M. leprae, the amplified copies as well as associated pseudogenes were absent (Table S4). Surprisingly, in a recent M. leprae genome, MRHRU-235-G from India (CP029543.1, direct submission), a tandem gene duplication was also noted.

The diguanylate cyclase/phosphodiesterase is a well-studied enzyme in diverse bacteria, harboring three domains: GAF as sensor domain, GGDEF as diguanylate cyclase, and EAL as c-di-GMP specific phosphodiesterase (38). The latter are two antagonistic enzymes, hence bifunctional, involved in the synthesis and hydrolysis of the second messenger cyclic di-GMP. The presence of three copies of its gene hints more c-di-GMP metabolism in *M. lepromatosis* for survival and/or pathogenicity in view that deletion of this gene in M. tuberculosis affects its dormancy and pathogenicity (39, 40). In M. leprae, studies have also suggested a potential role of diguanylate cyclase in the signaling response for intracellular survival (41). In other pathogens, a strategy has been designed to intercept c-di-GMP signaling pathways by directly targeting this second messenger as a way of controlling pathogen survival, growth or biofilm formation (42).

The gene *hemW*, previously annotated as *hemN* (10), was found in *M. lepromatosis* (MLPF_2234) but not in M. leprae. This gene likely encodes a heme chaperone that catalyzes the insertion of heme into hemoproteins for respiration (43, 44). The gene and adjacent MLPF_2235 (a pseudogene) stood between syntenic blocks 19 and 20 (Table S4); in M. leprae, however, the syntenic blocks rearranged afar after divergence, during which *hemW* and adjacent pseudogene (together ~2 kb) were likely lost. In M. haemophilum and M. tuberculosis, this gene was B586_09185 and Rv2388c, respectively, and their corresponding amino acids shared 89% and 81% identities with *M. lepromatosis*. This finding warrants further research into the function of *hemW*.

The two leprosy bacilli showed 6 genes in common to encode TetR family transcriptional regulators (*ethR* and others), and there were 5 additional ones in M. leprae but only pseudogenes in *M. lepromatosis*. These regulators are involved in diverse functions, such as regulation of c-di-GMP, ethionamide resistance, lipogenesis, etc. (45, 46). The difference in these regulators, along with many other unique genes, suggest considerable variations between the leprosy bacilli in metabolism, stress response, and interaction with the host to influence pathogenesis.

### Conclusions.

The history of the leprosy bacterial lineage started around 30 Mya when a mycobacterial ancestor diverged from the lineage of M. haemophilum. At that time, this ancestor would have a similar lifestyle to the present bacterium M. haemophilum, perhaps being able to produce opportunistic skin infections (12). Soon after this point, two lineages evolved, one leading to *M. uberis*, a pathogen infecting cows and goats (22) and the other leading to the MRCA of M. leprae and *M. lepromatosis*. Based on the phylogenies and pseudogene comparisons, the lineages of *M. uberis* and the MRCA likely underwent independent processes of reductive genome evolution (22). While the two leprosy lineages diverged around 13.9 Mya (10), our estimation of the ages of pseudogenes suggest that most of them formed shortly before the divergence. Afterwards, additional events of pseudogenization took place independently in both lineages, but at a much lesser scale.

We proposed previously that the reductive evolution of leprosy bacilli began in the MRCA as a parasite within the host, family *Hominidae* (great apes), ~18 to 20 Mya (11). In another word, the specific taming-adapting process has persisted all along until modern humans today. Our current genomic study refines the bacterial reductive evolution to ~14 Mya, which brings the host more specifically within subfamily *Homininae* (humans, gorillas, and chimpanzees). The genome reduction of *M. uberis* that also signifies a host specific process lends further support. Likewise, genome reduction of Mycobacterium lepraemurium from ancestral M. avium is likely unique to mice in causing murine leprosy (47).

Human M. leprae strains have been reported to infect a few animal species, such as nine-banded armadillos in the southern United States (8, 48) and nonhuman primates (48, 49). In the isolated infections of British red squirrels, the M. leprae strains were of human origin by hundreds of years while the *M. lepromatosis* strains bore a divergence of ~27,000 years from Mexican strain Mx1-22A (21). In red squirrels from continental northwest Europe, a recent study didn’t find such infections (50). Therefore, considering the finding of *M. lepromatosis* in Asian patients with leprosy (17, 18) and the likely introduction of the agent into Mexico 13,000 years ago during the earliest human Asia-Alaska-America migration (14), the most likely source of this bacillus in the British red squirrels could also be migrant humans from Asia ~27,000 years ago. In this regard, future studies on *M. lepromatosis* strains from Asian patients would be confirmative. Together, the new data and literature further corroborate our proposal.

The two leprosy bacilli share 71.4% of the genome but differ in 28.6%. The shared features should account for the dermal and neuronal invasion – the hallmark of leprosy. The preferential retention of PGL-I and its genes, favorable to bacterial survival, but inactivation of the detrimental PE/PPE components, are consistent features. Conversely, the variable genome features, in combination with the host genetic factors in diverse populations, should explain the variations in clinical and pathological manifestations of each infection, such as DLL and vascular occlusion associated with *M. lepromatosis*. Examples include three copies of the bifunctional diguanylate cyclase/phosphodiesterase gene in *M. lepromatosis* but one copy in M. leprae, 6 genes encoding the TetR transcriptional regulators in *M. lepromatosis* but 11 such genes in M. leprae, the presence of *hemW* in *M. lepromatosis* but absence in M. leprae, and so on. Clearly, the large numbers of shared and unique genome features require far more studies to delineate their relations with the infection. To this end, the annotated complete genome of *M. lepromatosis* has laid a foundation. In addition, recent report of passage of *M. lepromatosis* in mouse footpad (16) may provide viable bacteria for more studies as well as possible animal models in the near future.

The present study of the complete genome of *M. lepromatosis* also adds to the general knowledge on reductive genome evolution. This has been well documented in several bacterial endosymbionts and some other pathogens (51–54), involving a shift in lifestyle from relative free-life to host restriction and intracellular living. These genomes undergo chromosomal instability, rearrangements, loss of dispensable genes through either pseudogenization by point mutations or large deletions, and a tendency for genome downsizing. In general, the rates of nucleotide substitution in CDS also increase drastically, except a few examples of low rates in some long-term endosymbionts (55–58). Similar processes of massive pseudogenization have been detected in recent insect-associated bacterial symbionts due to their rich and stable new niches (59–61).

## MATERIALS AND METHODS

### Source of DNA and genome construction.

The *M. lepromatosis* DNA from strain FJ924 was extracted from a smear slide that was prepared from autopsy liver tissue of a patient originally from Mexico (4). The smear had been dried, acid-fast stained (Kinyoun method) for microscopy, and archived for 6 years (2007–2013). Total genomic DNA was obtained with the QIAamp kit (Qiagen, Valencia, CA) following instructions from manufacturer. The extraction yielded ~3 ng DNA. A whole-genome library was then constructed using the KAPA kit (Kapa Biosystems, Wilmington, MA). The library was enriched by six PCR cycles, quantified, assessed for size distribution, and sequenced on the HiSeq 2000 sequencer with the 75-bp pair end configuration (Illumina, San Diego, CA). A total number of 69 million reads were obtained.

The construction involved several steps. Human DNA contamination was removed first (14 million reads). From the remaining 55 million reads, mycobacterial DNA (11 million reads) were collected through BLAST matches (62) with M. leprae Br4923, filtering mostly contaminant DNA (44 million reads) from diverse other bacteria. The specific reads were assembled *de novo* using Velvet v1.2.10 (63), and the assembled contigs were aligned manually as well as using Bowtie v2.1.0 (64) to M. leprae template to build the first draft *M. lepromatosis* FJ924 genome. This draft was refined with GapFiller v1-10 (65) for gap closure and extension of contig edges, leading to announcement of a draft genome of 3,215,823 nucleotides with 39 contigs (66).

The published draft genome was used to map reads again, resulting in 12 million reads. These reads were assembled *de novo* with MIRA v2.1 with default parameters (67), and alignment of the assembled contigs led to third draft. The process of mapping, assembly, Gap filling, and draft construction was reiterated 5 times until seal of all blunt-end gaps. Eventually, a high-quality draft genome from 12.3 million reads and 525× genome coverage, with 12 gaps that were all flanked by repetitive contigs, was obtained. Primers were designed at nonrepetitive regions of contig edges and PCRs along with Sanger sequencing of amplicons were used to close these gaps.

### Genome annotation.

The closed *M. lepromatosis* FJ924 genome was annotated independently with Basys (68), RAST web-serves (69), and Prokka v1.12 (70). Annotation results from the three methods were compared and mixed with BEACON v1.1 (71). Initial pseudogene prediction was based on Prokka annotation of adjacent CDS presenting the same annotation and a custom python script. In brief, the script uses LAST (72) alignments to detect CDS which are 75% shorter than a reference set of clustered proteomes at 95% amino acid identity (Table S8). Then, it mixes adjacent fragmented genes when they overlap with the same reference hit.

Pseudogenes annotations were revised exhaustively by using several types of BLAST analyses. The main one was based on BLASTX using the complete *M. lepromatosis* FJ924 genome sequence as a query against a protein database of M. haemophilum (E value 0.01). This revision produced length changes of several pseudogenes, fusion of others, and conversion of some initially annotated CDS to pseudogenes.

### Comparative analysis of *M. lepromatosis* strains FJ924 and Mx1-22A.

To examine the differences between the draft genome Mx1-22A and the complete genome of strain FJ924, the 126 contigs of Mx1-22A were queried to FJ924 in BLASTN searches (E value E-180). The results produced a complete alignment of 134 shared segments. The noncovered segments of FJ924 were identified and their annotation features were extracted (for those higher than 300 bp) (Table S1). SNPs between the two strains were identified with MAUVE (23). The initial SNP number of 314 was reduced to 160 after removing ambiguous nucleotides in the contigs of Mx1-22A. In the second round, all inaccuracies and SNPs within the end 500 nucleotides of the contigs were removed, leaving behind a set of 17 SNPs. These SNPs were checked, and six of them were removed due to their location in a short segment with many ambiguous nucleotides. A final set of 11 SNPs were studied.

### Phylogenetics.

Several available mycobacterial proteomes were selected for phylogenetic reconstruction. OrthoFinder v2.3.3 (27) was used to detect, align, and concatenate 1,220 single core orthologous clusters of proteins shared between *M. lepromatosis* FJ924, M. leprae Br4923, M. haemophilum DSM44634, M. tuberculosis H37Rv and variant *bovis* AF2122/97, *M. uberis* Jura, M. marinum M, M. ulcerans SGL03, M. avium 104 and subsp. *paratuberculosis* K-10 (mafft aligner and IQ-TREE options) (27, 73, 74). Obtained concatenated alignment was pruned with Gblocks v0.91b (75) with the option *no gaps allowed*. A total of 316,716 amino acid positions from 418,308 were maintained and used for phylogenetic reconstruction. The species tree was inferred by the Maximum Likelihood method (JTT+F+R3 model) using IQ-TREE v1.6.12 with 1000 ultrafast bootstrap and SH-aLRT as node support (74). An additional phylogenetic tree was inferred by the Maximum Likelihood method (JTT+G+I+F model and 500 bootstrap replicates) for 15 diguanylate cyclase/phosphodiesterase proteins of several mycobacterial species (601 sites) in MEGA7 (76).

A time tree was inferred by applying the RelTime method (77) using the previous concatenated alignment and phylogenetic tree (316,716 sites and 10 sequences), a model JTT+G+I+F and branch lengths estimation by the ordinary least-squares approach in MEGA11 (78). The calibration constraint was the minimum and maximum time of the node of divergence between the two leprosy bacilli (8.2–21.4 Mya) as previously reported (10).

### Synteny and chromosomal rearrangement analysis.

Alignment of several mycobacterial genomes was performed with MAUVE (23) using default parameters. GenoPlotR was used to plot Mauve results (79). The permutation file was converted with the program GRIMM in a distant matrix, which contained the minimal number of inversions required between a pair of genomes to explain differences in gene order (26). Distance matrix was charged in MEGA7 (76) and a neighbor joining algorithm was used to infer the rearrangement phylogeny (80). Orthology between CDS and pseudogenes from M. leprae Br4923 and *M. lepromatosis* FJ924 was obtained with a reciprocal BLASTN best hit strategy (E value = 1.0E-05). Nonreciprocal hits and those hits in disagreement with gene order were revised upon visual inspection. Synteny was the first criterion to assign orthology when more than one similar hit was detected allowing up to 5 hits in new BLASTN searches.

### Identification and analysis of repetitive sequences.

A BLASTN search using the genome of *M. lepromatosis* as both query and subject with an E value of 10^−20^ was performed to search for repetitive sequences. Segments with more than one overlapping hit were revised to produce the repeat segment with the largest length. Repetitive sequences of the same family were aligned in Ugene v33.0 (81). To determine the effect of repeat segments over chromosomal rearrangements, the 24 synteny breaks were delimited by the last CDS/pseudogene in a block and the first CDS/pseudogene in the next block with an orthologous in the genome of M. leprae. A plot comparing repetitive sequences and synteny breaks was made with Circos (82).

### Identification of orthologous clusters.

Orthologous clusters of proteins (genes) in the forms of core, pan, pairwise shared, and strain specific clusters were obtained with OrthoFinder v2.3.3 using the *msa* (mafft aligner) and IQ-TREE options (27, 73, 74). Data on the clusters were used for several analyses.

### Estimation of the ages of pseudogenes.

The relative ages of M. leprae and *M. lepromatosis* pseudogenes were estimated according to the method of Gomez-Valero et al. (7) with inclusion of the recent M. haemophilum genome data (12) for closer phylogeny. Briefly, this method used M. tuberculosis as outgroup to root the tree and compared M. haemophilum with either M. leprae or *M. lepromatosis*. The divergence between the two species determines the relative age of pseudogenization, ranging from 1, when it took place at the start of the divergence, to 0 at this time. The method is based on the estimation of the dN and dS (the number of nonsynonymous and synonymous substitutions per site, respectively) for each cluster of orthologous gene-pseudogene in the branches leading to M. haemophilum and a leprosy bacillus.

Estimation of the dN and dS values required use of clusters of orthologous genes. To produce clusters of orthology that incorporated M. leprae and/or *M. lepromatosis* pseudogenes and corresponding genes from both M. haemophilum and M. tuberculosis, a reciprocal best hit strategy was used with E value 1.0E-05. The set of pseudogenes/genes of *M. lepromatosis* and M. leprae were then compared, and they were further aligned to the sets of M. haemophilum and M. tuberculosis.

Alignments of genes and pseudogenes to maintain the codon structure, despite the indel mutations of the pseudogenes, were performed with MACSE (83). This program was also used to align clusters of functional genes in the four species. The program codeML (84) was used to obtain the dN and dS values as previously described (58). A custom python script was used to run those analyses.

To estimate relative pseudogene ages (*Rpa*), the following formulas were used, for *M. lepromatosis* (up) and for M. leprae (down):

$$\text{Rpa}=({\text{dN}}_{{\text{ilp}}_{\text{ps}}}-f(\text{dNih}))/(\bar{{\text{dSilp}}_{\text{ps}}}-f(\text{dNih}))$$

$$\text{Rpa}=({\text{dN}}_{{\text{ile}}_{\text{ps}}}-f\prime (\text{dNih}))/(\bar{{\text{dSile}}_{\text{ps}}}-f\prime (\text{dNih}))$$

The i (intercept) corresponds to the node of divergence between the leprosy bacilli and M. haemophilum lineages. The values dN_ilp_ps_ and dN_ile_ps_ are the numbers of nonsynonymous substitutions in the branches of a pseudogene from *M. lepromatosis* and M. leprae, respectively. To be pointed out, the term dN only shows a strict sense for the period of evolution as a gene.

The parameters f(dN_ih_) and f’(dN_ih_) are estimates, for orthologous genes, of the expected dN value for the branch of a leprosy bacillus as a function of the dN value for the branch of M. haemophilum considering that this gene had been evolving as a functional gene for the complete period of time. Orthologous genes evolve faster in the leprosy bacilli than in M. haemophilum, especially for dN (four times faster on average). By using dN values of 1,213 clusters of orthologous genes, the relationship between outcome and predictor, both log-transformed, was estimated by segmented linear regression. A two-piece linear relationship was assumed, namely, represented by two straight lines connected at an unknown breakpoint. Estimation was performed by means of Muggeo's algorithm (85) as implemented in the library *segmented* of the R software (version 4.0.4) (86). The last term of the formula, the average dS in the branch leading to *M. lepromatosis* and M. leprae pseudogenes, may be considered a value close to the number of substitutions per site in a nonfunctional DNA sequence (0.2851 and 0.2813 for *M. lepromatosis* and M. leprae, respectively). These are the expected dN values for pseudogenes with *Rpa* values of 1.

The time of divergence in Mya between the lineage of leprosy bacilli and the M. haemophilum lineage (*Rpa *= 1) was estimated with the RelTime method (28). All mentioned scripts are available at https://github.com/DiegoSantos-Garcia/Scripts.

### Data availability.

This whole-genome shotgun project has been deposited at DDBJ/EMBL/GenBank under the accession number CP083405 and BioProject PRJNA281005.
